# Drug Resistance Profile and Clinical Features for Hepatitis C Patients Experiencing DAA Failure in Taiwan

**DOI:** 10.3390/v13112294

**Published:** 2021-11-17

**Authors:** Chun-Ming Hong, You-Yu Lin, Chun-Jen Liu, Ya-Yun Lai, Shiou-Hwei Yeh, Hung-Chih Yang, Jia-Horng Kao, Shih-Jer Hsu, Yi-Hsiang Huang, Sheng-Shun Yang, Hsing-Tao Kuo, Pin-Nan Cheng, Ming-Lung Yu, Pei-Jer Chen

**Affiliations:** 1 Division of Hospital Medicine, Department of Internal Medicine, National Taiwan University Hospital, Taipei 10022, Taiwan; thomashong0218@gmail.com; 2 Graduate Institute of Clinical Medicine, National Taiwan University College of Medicine, Taipei 10002, Taiwan; youylin@ntu.edu.tw (Y.-Y.L.); cjliu@ntu.edu.tw (C.-J.L.); kaojh@ntu.edu.tw (J.-H.K.); 3 Division of Gastroenterology, Department of Internal Medicine, National Taiwan University Hospital, Taipei 10022, Taiwan; hcyang88@ntu.edu.tw (H.-C.Y.); shihjer.hsu@gmail.com (S.-J.H.); 4 Center of Precision Medicine, National Taiwan University, Taipei 10055, Taiwan; yayun1022@hotmail.com (Y.-Y.L.); shyeh@ntu.edu.tw (S.-H.Y.); 5 Graduate Institute of Microbiology, National Taiwan University College of Medicine, National Taiwan University, Taipei 10051, Taiwan; 6 Department of Internal Medicine, National Taiwan University Hospital Yunlin Branch, Yunlin 64000, Taiwan; 7 Division of Gastroenterology and Hepatology, Department of Internal Medicine, Taipei Veterans General Hospital, Taipei 11217, Taiwan; yhhuang@vghtpe.gov.tw; 8 Institute of Clinical Medicine, National Yang Ming Chiao Tung University School of Medicine, Taipei 11230, Taiwan; 9 Division of Gastroenterology and Hepatology, Department of Internal Medicine, Taichung Veterans General Hospital, Taichung 40705, Taiwan; yansh2525@gmail.com; 10 Chi Mei Medical Center, Department of Internal Medicine, Tainan 71004, Taiwan; kuohsingtao@gmail.com; 11 Department of Internal Medicine, National Cheng Kung University Hospital, Tainan 70403, Taiwan; pncheng@mail.ncku.edu.tw; 12 Department of Internal Medicine, Kaohsiung Medical University Hospital, Kaohsiung Medical University, Kaohsiung 80756, Taiwan

**Keywords:** direct-acting antiviral agent, chronic hepatitis C, treatment failure, resistance-associated substitution, whole genome sequencing, Taiwan

## Abstract

About 4% of the population in Taiwan are seropositive for anti-HCV Ab and 70% with HCV RNA. To address this high chronic hepatitis C disease load, Taiwan National Health Insurance started reimbursing genotype-specific DAAs in 2017 and pangenotype DAAs in mid-2018. With a 97% SVR12 rate, there were still 2–3% of patients that failed to clear HCV. To understand the causes of DAA failure in Taiwan, we conducted a multi-center, clinical, and virologic study. A total of 147 DAA-failure patients were recruited, and we searched HCV NS3/4A, NS5A and NS5B for known resistance-associated substitutions (RASs) by population sequencing, and conducted whole genome sequencing (WGS) for those without known RASs. A total of 107 patients received genotype-specific DAAs while 40 had pangenotype DAAs. Clinically, the important cause of failure is poor adherence. Virologically, common RASs in genotype-specific DAAs were NS5A-L31, NS5A-Y93, and NS5B-C316, while common RASs in pangenotype DAAs were NS5A-L31, NS5A-A/Q/R30, and NS5A-Y93. Additionally, new amino acid changes were found by WGS. Finally, we identified 12 cases with inconsistent baseline and post-treatment HCV genotypes, which is suggestive of re-infection rather than treatment failure. Our study described the drug resistance profile for DAA failure in Taiwan, showing differences from other countries.

## 1. Introduction

Viral hepatitis has posed significant global health burden and is responsible for an estimated 1.4 million deaths per year from acute hepatitis to chronic hepatitis-related liver cancer and cirrhosis—a toll comparable to that of HIV and tuberculosis. Among these deaths, approximately 47% are attributable to hepatitis B virus, 48% to hepatitis C virus, and the remainder to hepatitis A virus and hepatitis E virus. Therefore, eliminating viral hepatitis is a major sustainable development goal (SDG) set by the WHO (World Health Organization). WHO is working on global targets to reduce new viral hepatitis infections by 90% and reduce deaths due to viral hepatitis by 65% by 2030 [1]. To address these SDG, vaccinations become the major method for control of hepatitis A, B, and E, but for hepatitis C, treatment is the mainstream.

Chronic hepatitis C virus (HCV) infection is associated with progressive liver disease, which can lead to liver cirrhosis and hepatocellular carcinoma (HCC). Globally, an estimated 71 million people have chronic hepatitis C infection. Since the discovery of HCV in the late 1980s, six genotypes have been identified, in which the most prevalent genotype is genotype 1b, accounting for more than 50% of all HCV infections [2,3,4], followed by genotype 2 [5]. Currently, no vaccine is available for hepatitis C infection. However, with the introduction of potent all-oral direct-acting antiviral agents (DAAs), the treatment of chronic hepatitis C (CHC) has entered a new era of curative therapy.

**Figure 1:**
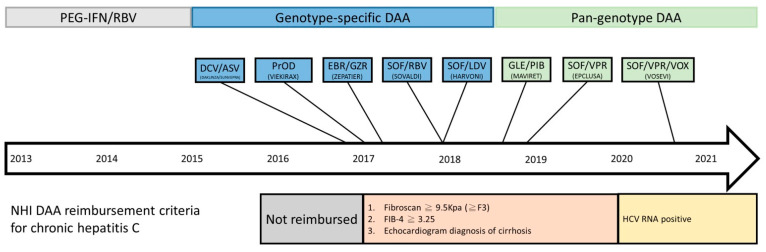
Timeline of DAA reimbursement in National Health Insurance (NHI) in Taiwan.

In Taiwan, the estimated prevalence of HCV is 3.28% (1.8–5.5%) in the general population, and even more than 10% in some hyperendemic areas [6,7]. To effectively control and treat chronic hepatitis C, the Taiwan National Health Insurance Bureau started interferon and ribavirin combination therapy in 2004, but phased into reimbursing DAAs in year 2017. As in other countries, the DAA reimbursement in Taiwan began with priority patients, only for those who failed previous interferon and ribavirin therapy and with fibrosis stage above F3. Later in 2017, DAAs were reimbursed for patients whose fibrosis stage was above F3, regardless of treatment experience. In September 2019, DAAs were reimbursed for all chronic hepatitis C patients. The introduction of different DAAs also evolved in the past several years. Since January 2017, the earliest available DAAs were daclatasvir/asunaprevir and dasabuvir plus ombitasvir/paritaprevir/ritonavir. Then in August 2017, elbasvir/grazoprevir was introduced. In 2018, sofosbuvir and sofosbuvir/ledipasvir were added. Then glecaprevir/pibrentasvir was included later. Since June 2019, sofosbuvir/velpatasvir was reimbursed. Genotype-specific DAA era transitioned into pangenotype-DAA era after August 2018 in Taiwan (Figure 1). In order to learn the effectiveness and barriers for DAA therapies, Taiwan Association for the Study of the Liver (TASL) established a Taiwan HCV Registry (TACR) in July 2020 to recruit DAA-experienced subjects and their clinical histories. Till year 2021, a total of 13951 patients from 48 study sites participated in the program and provided useful clinical profiles about DAA therapy in Taiwan [8]. 

DAAs that have been approved in Taiwan over the years include sofosbuvir, sofosbuvir/ledipasvir, daclatasvir/asunaprevir, dasabuvir plus ombitasvir/paritaprevir/ritonavir, elbasvir/grazoprevir, sofosbuvir + daclatasvir, glecaprevir/pibrentasvir, and sofosbuvir/velpatasvir. Among these regimens, sofosbuvir/velpatasvir, sofosbuvir + daclatasvir, and glecaprevir/pibrentasvir are pangenotypic whereas sofosbuvir, sofosbuvir/ledipasvir, daclatasvir/asunaprevir, dasabuvir plus ombitasvir/paritaprevir/ritonavir, and elbasvir/grazoprevir are genotype-specific. DAAs act on non-structural NS3/4 protease, NS5A, and NS5B to stop viral replication. Compared to pegylated-IFN and ribavirin, DAAs are highly effective with SVR12 (sustained virologic response) rate over 90% in general. 

However, from another perspectives, about 3–5% of CHC patients do not achieve SVR despite completion of DAA therapy. The case number of this DAA failure patients increased significantly, as DAA regimens roll-out to more and more CHC patients. The main causes of DAA failure consist of host factors and virologic factors. Host factors include liver disease stages, comorbidities, or poor compliance, while virologic factors include genotype, viral load, and the presence of resistance-associated substitutions (RASs) [9]. In HCV genome, the presence of many RASs at specific sites have caused viral resistance to DAAs [10,11]. It is noteworthy that single RAS may be observed in DAA-naive patients to some extent [12,13,14,15,16], though dual RASs are extremely rare [17,18]. Baseline RASs attenuate the efficacy of DAAs. Meanwhile, some RASs are generated after the failure of DAA treatment. Furthermore, HCV reinfection is a unique challenge among high risk CHC patients receiving curative DAAs [19].

The prevalence of RASs can be an important information for clinicians when choosing appropriate rescue DAA regimens after DAA failure. Therefore, our study investigated the clinical features of DAA failures, and the nature of RASs after failure of DAA therapy in Taiwan and aimed to find out some other mutant substitutions that are potentially related to drug resistance, and proposed appropriate new DAAs for rescue therapy.

## 2. Materials and Methods

### 2.1. Patients

In total, 21 hospitals and one clinic across Taiwan joined our study and 147 DAA failure patients were recruited from January 2019 to December 2020. Each patient’s medical records were reviewed, and data were extracted and validated using a standardized case report form and a unified coding dictionary. Eligible patients were those who (1) were aged >20 years, (2) had detectable HCV RNA, and (3) received DAA regimes for at least one dosage of any DAA and had treatment outcomes available. Clinical information such as age, gender, HCV genotype, DAA regimen, comorbidity, history of hepatocellular carcinoma, and laboratory data (aspartate trans-aminase (AST), alanine aminotransferase (ALT), platelet count, and HCV RNA load) as well as serum samples were obtained. The treatment regimens and strategies conformed to the regulations of the Health and Welfare Department of Taiwan and regional guidelines [8,20]. Treatment failure was defined as virological failure either by, incomplete treatment, premature discontinuation with adverse events, viral breakthrough, or relapse after completion of treatment. 

Given the study design, we were unable to obtain serum samples before DAA treatment and lack for control cohort. Considering TACR cohort is the nationwide registry platform, we chose TACR cohort as the control of our study.

### 2.2. Identification of RASs

Direct sequencing was used to detect RASs in NS3, NS5A, and NS5B regions of the HCV genome. We established PCR (polymerase chain reaction) platform for these non-structural proteins and searched the associated mutant genes by population sequencing via the Sanger method (Table S1). The limit of detection of HCV RNA for RAS in our laboratory was 5000 cp/mL. Analyses of RAS were not eligible in 11 patients because of low viral load. In the meantime, we performed whole genome sequencing for cases without known RASs. Amplicon-based whole genome sequencing was performed by amplification and Sanger sequencing of overlapping amplicons covering the whole HCV genome (Tables S2–S9). 

### 2.3. Preparation of HCV RNA

HCV RNA was extracted from plasma sample using MagNA Pure LC Total Nucleic Acid Isolation kit (Roche Diagnostics Applied Science, Basel, Basel-Stadt, Switzerland) by the manufacture’s instruction. HCV RNA was subjected to reverse transcription using the SuperScript^®^ III Reverse Transcriptase (Invitrogen, Waltham, MA, USA) with random hexamer at 65 °C for 5 min, 4 °C for 2 min, and incubating the reaction mixture at 25 °C for 15 min, 50 °C for 50 min, and 85 °C for 5 min. The resulting cDNA was used as a template for PCR amplification.

### 2.4. Quantification of HCV cDNA by Real-Time PCR

HCV cDNA was processed to a LightCycler based real-time PCR assay with fluorescent hybridization probes (Roche Diagnostics Applied Science). The primer set used for PCR amplification is 5′-GAG GAA CTA CTG TCT TCA CG-3′ (forward primer at nt. 49–68) and 5′-GTT GAT CCA AGA AAG GAC CCG GTC-3′ (reverse primer at nt. 184–207). The probes used for quantification are 5′-AGC CAT AGT GGT CTG CGG AAC C-FLU-3′ (anchor probe at nt. 136–157) and 5′LC-Red640-TGA GTA CAC CGG AAT IGC IAG GA-PH3′ (sensor probe with nt. at 160–182). The fluorescence-labeled hybridization probes were synthesized by TIB MOLBIOL. Thermal cycling conditions were as follows: initial denaturation at 95 °C for 10 min, followed by 45 cycles of denaturation at 95 °C for 10 s, annealing at 51 °C for 5 s, extension at 72 °C for 14 s. 

### 2.5. Two-Step Nested PCR for Resistance Mutation Analysis

Two-step nested PCR was carried out with primers specific for the NS3, NS5A, and NS5B region of the HCV genome. Three microliters of HCV cDNA was subjected to the first run of PCR. Of the first run, 0.2 µL of product was subjected to the second run of PCR. Each PCR contained 10× PCR buffer, 2 mM magnesium chloride, 0.16 mM dNTP, 0.8 µM of forward and reverse primers and 0.2 µL of Platinum^®^ Taq DNA Polymerase (Invitrogen) in a total volume of 20 µL.

### 2.6. Statistical Analysis 

The prevalence of RASs in different groups was described. Chi-square and Fisher’s exact test were used to search for factors responsible for differences in the rate of RAS prevalence and profile; a *p*-value of <0.05 was considered as significant. Significance between prevalence of RASs after failure of regimens was analyzed using a chi-square test. All statistical analyses were performed with Windows Excel or R.

## 3. Results

All the patient characteristics and comparison with those from TACR cohort are shown in Table 1. Among the 147 enrolled patients in our study, 77 (54%) patients were male, the average age was 60 years, and 22% were treatment-experienced. Previous treatments included mostly pegylated-interferon (PEG-IFN)/ribavirin (RBV), and some daclatasvir/asunaprevir and sofosbuvir/ledipasvir. Five percent were coinfected with HBV (hepatitis B virus) and about 6% were coinfected with HIV (human immunodeficiency virus). Thirty-five percent were cirrhotic and 16% had active hepatocellular carcinoma. The mean pre-treatment Fib-4 was 3.86. About 30% were genotype 1b while more than 36% were genotype 2. Regarding DAA regimens, sofosbuvir/ledipasvir and glecaprevir/pibrentavir were the top two commonly-used DAAs. While in the TACR cohort, 236 patients were DAA-failure cases. Among them, 49% were male, and the average age was 63 years. Genotype 1 (47%) was the most, followed by genotype 2 (45%). 

The overall clinical features of our study subjects were similar to those of the larger TACR cohort but with certain distinctions. For instance, the percentages of coinfection of HIV and people who inject drugs (PWID) were significantly higher in our cohort, while the percentages of chronic kidney disease (CKD), liver cirrhosis, and early termination were significantly higher in the TACR cohort. The percentage of glecaprevir/pibrentasvir was significantly higher in our cohort while the percentage of sofosbuvir/ribavirin was higher in TACR cohort. With regard to virus features, the percentage of genotype 6 was significantly higher in our cohort than in the TACR cohort. 

Furthermore, these 147 patients can be categorized into two groups: one group (107 patients) received genotype-specific DAAs while the other one (40 patients) received pangenotype DAAs. Sofosbuvir/velpatasvir, glecaprevir/pibrentasvir, and sofosbuvir/daclatasvir are pangenotypic whereas the earlier DAAs such as sofosbuvir, sofosbuvir/ledipasvir, daclatasvir/asunaprevir, dasabuvir plus ombitasvir/paritaprevir/ritonavir, and elbasvir/grazoprevir are genotype-specific. The clinical characteristics of these two groups are again shown in Table 1. With regard to clinical features, the percentages of liver cirrhosis and patients with prior treatment were significantly higher in genotype-specific DAA group. Regarding virus features, genotype 1b was the most-common genotype in genotype-specific DAA group, while genotype 2a was the most-common genotype in pangenotype DAA group. In addition, the percentages of genotype 3 and genotype 2a were significantly higher in pangenotype DAA group, while the percentage of genotype 1b was significantly higher in genotype-specific DAA group. These differences may reflect the evolution of relaxing reimbursement criteria to recruit more diverse CHC patients, and the perception/enthusiasm about DAA therapies in CHC patients during this period. Furthermore, we noticed some off-label use in some genotype-specific DAAs. Six genotype 6 patients were treated with daclatasvir/asunaprevir, dasabuvir plus ombitasvir/paritaprevir/ritonavir, sofosbuvir/ribavirin, and elbasvir/grazoprevir, whereas a genotype 1b patient took sofosbuvir and ribavirin.

**Figure 2:**
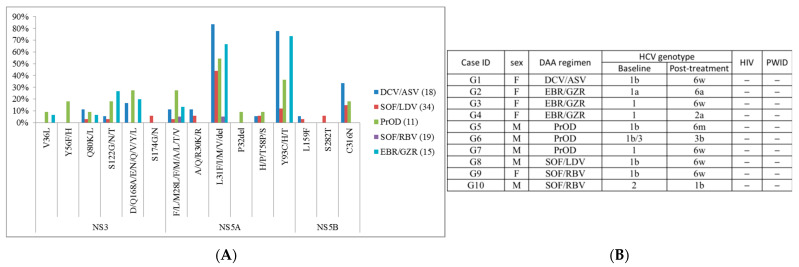
Summary of (**A**) RASs in patients with genotype-specific DAA treatment and (**B**) cases with different baseline/post-treatment HCV genotype. “−”, negative.

Details of genotype-specific DAAs are shown in Table S10. Summary of RASs of genotype-specific DAA group is shown in Figure 2A and Table S11. NS5A-L31, NS5A-Y93, and NS5B-316 were the most frequent RASs in genotype-specific DAA failure cases, while other detected RASs were observed at low frequencies. Ten cases were identified to have different post-treatment HCV genotype compared to its baseline HCV genotype, but none belonged to high-risk groups (HIV positive or PWID), and one case reported mixed genotypes at baseline (Figure 2B). 

**Figure 3:**
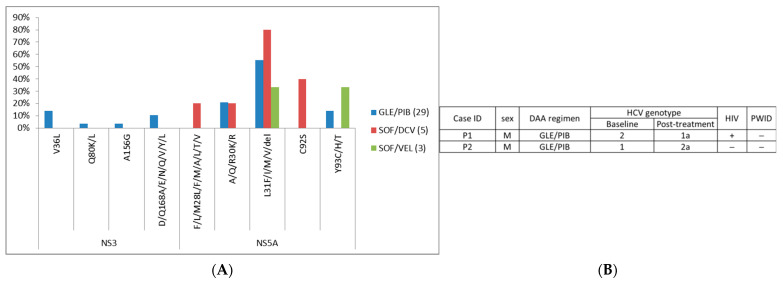
Summary of (**A**) RASs in patients with pan-genotype DAA treatment and (**B**) cases with different baseline/post-treatment HCV genotype. “+”, positive; “−”, negative.

Meanwhile, details of pangenotype DAAs are shown in Table S12. Summary of RASs of pangenotype DAA group is shown in Figure 3A and Table S13. NS5A-L31, NS5A-A/Q/R30, and NS5A-Y93 were the most frequent RASs in pangenotype DAA failure cases, while other detected RASs were observed at low frequencies. Two cases were identified to have different post-treatment HCV genotype compared to its baseline HCV genotype, and one case was reported as HIV positive (Figure 3B).

The most frequent RASs (NS5A-L31 and NS5A-Y93) were frequently observed in both genotype-specific and pangenotype DAA failure cases. RAS analysis was not available in some cases because of lack of serum sample or low viral titer. Therefore, the sample numbers in RAS results are equal or lower than those in clinical characteristics.

Moreover, for DAA failure patients with no known RASs identified in NS5A, NS5B, and NS3, we further conducted a whole genome sequencing to discover potential RASs as listed in Table 2. These potential RASs were identified in previously unsearched regions of NS3, NS5A and NS5B genes.

## 4. Discussion

Direct-acting antiviral agents (DAAs) have been reimbursed by National Health Insurance (NHI) in Taiwan since 2017, and the reimbursement criteria have changed several times as previously described. The annual adjustment of reimbursement criteria and available DAAs were important background for the interpretation of our study results. The timeline of reimbursement of DAAs in Taiwan is shown in Figure 1. With regard to clinical characteristics, the percentages of patients with prior treatment and liver cirrhosis were higher in the earlier genotype-specific DAAs possibly because of the reimbursement criteria. DAAs were reimbursed for patients with treatment experience and with severe fibrosis in the beginning. The restrictions were removed largely in the pangenotype DAA era. Meanwhile, the percentage of patients with genotype 1b was significantly higher in genotype-specific DAA era may be due to the available DAAs at that time. Among the genotype-specific DAAs, sofosbuvir/ledipasvir, daclatasvir/asunaprevir, dasabuvir plus ombitasvir/paritaprevir/ritonavir, and elbasvir/grazoprevir were aimed to treat genotype 1b.

In our study, off-label use is noted in some genotype-specific DAAs. Off-label use was reported sometimes in real-world practice especially in the early days of DAA era. This was because the conditions of clinical trials could not always apply to complex real-world practice. However, off-label use could be one of the reasons of DAA treatment failure.

Since our study recruited only DAA failure patients, it was not feasible to analyze the impact of clinical features to treatment failure. As shown in Table 1, we compared our cases with the failure cases from TACR cohort. The overall clinical features of our study subjects were similar to those of the larger TACR cohort in general with only certain distinctions. Yu et al. reported the most important factor independently associated with treatment failure was DAA adherence < 60% [21]. Therefore, the single clinical factor most importantly for successful DAA therapy is close monitoring of patient’s compliance [22]. Nonetheless, not so much literature was reported on poor compliance of DAA therapy. This important clinical cause of failure has to be carefully investigated and addressed in the future.

**Figure 4:**
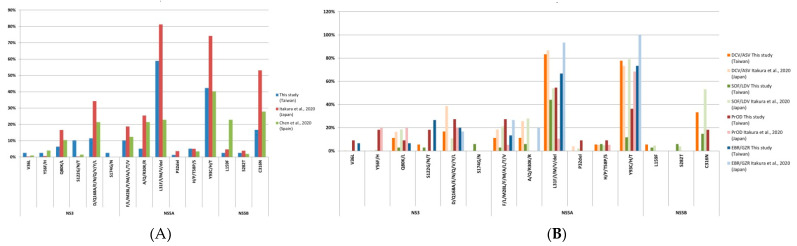
Comparison of RAS prevalence with other studies. (**A**) RAS prevalence compared to studies of Japan [23] and Spain [24]; (**B**) Detailed comparison of RAS prevalence for each DAA treatment to study results of Japan [23].

Our study assessed the prevalence of RASs in Taiwan and compared it with that in other countries as shown in Figure 4. Itakura et al. reported features of RASs in 1193 genotype 1b patients in Japan [23]. Similar to our study, NS5A-L31 and Y93 are the major RASs in Dr. Itakura’s study. However, the prevalence of NS3-S122 in our study is significantly higher (26%) while the prevalence of NS5A-R30 is significantly lower (0%). No difference is observed among NS5B RASs. Furthermore, our study comprises more HCV genotypes than the study from Dr. Itakura’s team.

In the meantime, Chen et al. reported features of RASs in 220 Spanish patients from 39 Spanish hospitals [24]. In comparison, the prevalence of NS5A-L31 in genotype 1b of our study (67%) is higher than that of the Spanish study (39%), whereas the prevalence of NS5B-L159F in genotype 1b (8%) is significantly lower than that of the Spanish study (57%). Different DAA regimens, HCV genotypes, or different periods of recruitment may attribute to these differences.

Among these DAA regimens, glecaprevir/pibrentasvir and sofosbuvir/velpatasvir are currently the principal regimens, whereas many regimens are no longer available or rarely used. Therefore, we specifically focus on the RASs on glecaprevir/pibrentasvir and sofosbuvir/velpatasvir. In our study, four patients took sofosbuvir/velpatasvir and thirty-one took glecaprevir/pibrentasvir. 

Among these 31 patients of glecaprevir/pibrentasvir treatment failure, 2a and 3b are the most common genotypes. The characteristics of these patients are shown in Table S12, although no conclusion can be made due to limited patient numbers. Adolfo de Salazar et al. reported the prevalence of RASs after failure of glecaprevir/pibrentasvir in Europe [25]. Among these 90 European patients, 31 patients (34.4%) failed glecaprevir/pibrentasvir without any NS3 or NS5A RASs, 62.4% (53/85) showed RASs in NS5A, 15.6% (13/83) in NS3 and 10% (9/90) in both NS5A and NS3. Infection with HCV genotypes 1a and 3a was found to be associated with a higher prevalence of NS5A RASs. 

In addition to detecting RASs in NS3/4A, NS5A, and NS5B in HCV genome, we did whole genome analysis for those without known RASs. We discovered some potential RASs in initially non-sequenced regions of NS3, NS5A, and NS5B. To our knowledge, these potential RASs identified by whole genome analyses have yet to be reported, and their functions during HCV infection are not clear. A larger sample size is required to further validate our current findings, but results have shown potential whole genome sequencing can provide for our further understanding of RAS drug resistance during HCV DAA treatment.

It is critical to distinguish HCV re-infection from true DAA treatment failure when studying DAA-related RASs, as both present with detectable HCV post-treatment but for different reasons. A total of 12 cases were identified in our study to have different HCV genotypes at baseline compared to post-treatment, which is suggestive of re-infection. Studies have reported higher HCV re-infection rate in HIV-positive and PWID patients. Baseline viral sample would be required to resolve whether the different HCV genotype between baseline and post-treatment resulted from re-infection, genotyping error, or mixed-infection.

However, there are some limitations to our study. We do not have serum samples before DAA therapy due to the study design. Therefore, it is unable to know whether these RASs exist already before DAA therapy or emerge during DAA therapy. In addition, the duration between end of treatment and acquisition of serum sample is not unanimous, and RASs will be gradually replaced by wild type. Moreover, NS3-RASs are known to disappear earlier than NS5A-RASs [26]. Third, most patients in our study are not high-risk groups for HCV infection and the reinfection rate should be low, there is still the possibility of some treatment failure cases are actually results of HCV reinfection. Lastly, the case number of whole genome analysis was not sufficient. We should increase the number of whole genome analysis to detect potential RASs. More importantly, the study did not address the rescue therapies for these DAA failure patients. Recently, sofosbuvir/ velpatasvir/voxilaprevir (Vosevi) was reimbursed in Taiwan and will become a main rescue regimen to be followed.

In conclusion, our study is the first one depicting drug resistance profile for DAA failure in Taiwan. We observed some differences from other countries. These can be important references for clinical practice as well as virologic studies. Meanwhile, whole genome sequencing can be another important method to discover some potential RASs although further studies are needed. Their relevance to future rescue therapy warrants investigation.

## Figures and Tables

**Table 1:** Summary of patient characteristics and comparison to TACR DAA treatment failure cases. Statistical comparisons (student *t*-test) were performed between genotype-specific and pan-genotype casesa and between the study cohort of this study and TACR failure casesb. *p*-value: *, <0.05; **, <0.01; ***, <0.001. DAA, direct-acting antiviral agents; SOF, Sofosbuvir; DCV, Daclatasvir; LDV, Ledipasvir; PrOD (PTV, Paritaprevir; r, Ritonavir; OBV, Ombitasvir; DSV, Dasabuvir); GZR, Grazoprevir; GLE, Glecaprevir; EBR, Elbasvir; PIB, Pibrentasvir; VEL, Velpatasvir; RBV, Ribavirin; ASV, Asunaprevir. HBV, hepatitis B virus; HIV, human immunodeficiency virus; CKD, chronic kidney disease; PWID, People who inject drugs; LC, liver cirrhosis; HCC, hepatocellular carcinoma. TACR, The Taiwan HCV Registry. n/a, not available.

	This Study			TACR (Chen et al.)
	Total	Genotype-Specific	Pan-Genotype	Failure Cases
Study population, *n*	147	107	40	236
Gender (male), *n* (%)	77 (54%)	53 (50%)	24 (67%)	115 (49%)
Age (years), mean ± SD	60 ± 13	61 ± 12	57 ± 14	63
HBV, *n* (%)	7 (5%)	4 (4%)	3 (9%)	16 (7%)
HIV, *n* (%)	8 (6%)	5 (5%)	3 (10%)	4 (2%) *
CKD, *n* (%)	8 (6%)	6 (6%)	2 (6%)	40 (17%) ***
PWID, *n* (%)	9 (7%)	5 (5%)	4 (13%)	3 (1%) **
LC, *n* (%)	45 (35%)	39 (40%)	6 (19%)*	104 (44%) **
Active HCC, *n* (%)	20 (16%)	16 (16%)	4 (13%)	23 (10%)
Pre-treatment FIB-4, mean ± SD	3.86 ± 3.19	4.09 ± 3.28	3.14 ± 2.71	n/a
With prior treatment, *n* (%)	31 (22%)	27 (27%)	4 (11%)*	64 (27%)
HCV genotype, *n* (%)				
1	3 (2%)	3 (3%)	0 (0%)	112 (47%)
1a	10 (7%)	7 (7%)	3 (8%)	
1b	43 (30%)	42 (40%)	1 (3%) ***	
2	5 (3%)	4 (4%)	1 (3%)	107 (45%)
2a	36 (25%)	20 (19%)	16 (41%) **	
2b	17 (12%)	14 (13%)	3 (8%)	
3a, 3b, 3k	8 (6%)	1 (1%)	7 (18%) ***	5 (2%)
6, 6a, 6n, 6n, 6w	21 (15%)	13 (13%)	8 (21%)	7 (3%) ***
DAA regimes, *n* (%)				
DCV/ASV	18 (12%)	18 (17%)	0 (0%)	24 (10%)
SOF/RBV	24 (16%)	24 (22%)	0 (0%)	61 (26%) *
SOF/LDV	34 (23%)	34 (32%)	0 (0%)	64 (27%)
PrOD	13 (9%)	13 (12%)	0 (0%)	34 (14%)
EBR/GZR	18 (12%)	18 (17%)	0 (0%)	29 (12%)
SOF/VEL	4 (3%)	0 (0%)	4 (10%)	5 (2%)
GLE/PIB	31 (21%)	0 (0%)	31 (78%)	20 (8%) ***
SOF/DCV	5 (3%)	0 (0%)	5 (13%)	5 (2%)
DAA termination, *n* (%)	2 (1%)	2 (2%)	0 (0%)	21 (9%) **

**Table 2:** Potential RASs identified by whole genome sequencing (WGS).

Genotype	Gene	Potential RASs
1b	NS3	M615V
2b	NS5B	V138I
		R563G
		O565R
3b	NS3	S224A
		V386I
		A402T
		A451T
	NS5A	A103T
		A197T
		A350T
		A401T

## Data Availability

The data that support the findings of this study are available on request from the corresponding author. The data are not publicly available due to privacy or ethical restrictions.

## Supplementary Materials

The following are available online at https://www.mdpi.com/article/10.3390/v13112294/s1, Table S1: Primers for population sequencing of HCV NS3, NS5A, and NS5B, Table S2: Primers for whole genome sequencing of HCV genotype 1a, Table S3: Primers for whole genome sequencing of HCV genotype 1b, Table S4: Primers for whole genome sequencing of HCV genotype 2a, Table S5: Primers for whole genome sequencing of HCV genotype 2b, Table S6: Primers for whole genome sequencing of HCV genotype 3b, Table S7: Primers for whole genome sequencing of HCV genotype 6a, Table S8: Primers for whole genome sequencing of HCV genotype 6n, Table S9: Primers for whole genome sequencing of HCV genotype 6w, Table S10: Summary of patient characteristics for genotype-specific DAA patients, Table S11: Summary of RASs in patients with genotype-specific DAA treatment, Table S12: Summary of patient characteristics for pangenotype DAA patients, Table S13: Summary of RASs in patients with pangenotype DAA treatment.
